# Study of Acid-Base Disorders and Biochemical Findings of Patients in a Tertiary Care Hospital: A Descriptive Cross-sectional Study

**DOI:** 10.31729/jnma.4722

**Published:** 2019-12-31

**Authors:** Rupesh Kumar Shreewastav, Krishna Prasad Jaishi, Madan Raj Pandey, Ganesh Prasad Singh, Arambam Giridhari Singh

**Affiliations:** 1 Department of Biochemistry, Nobel Medical College Teaching Hospital, Biratnagar, Nepal

**Keywords:** *acidosis*, *alkalosis*, *critical illness*, *pH*

## Abstract

**Introduction:**

Acid base disorder is a condition characterized by alteration in blood pH by the imbalance between the components of blood leading to a life threatening situation. The main aim of this study was to find the prevalence of acid-base disorders and biochemical findings of such disorders in patients in a tertairy care hospital.

**Methods:**

This descriptive cross-sectional study was conducted in Nobel Medical College Teaching Hospital from 1^st^ September, 2018 to 31^st^ August, 2019. Ethical apporoval was taken from Institutional Review Committee. All the patients presented to emergency department, intensive care units and wards were included during the study period. Data were entered and calculations were done in Microsoft Excel, point estimate at 95% Confidence Interval was calculated along with frequency and proportion for binary data.

**Results:**

Out of arterial blood gas analysis of 1144 patients, the prevalence of acid base disorders was 718 (62.76%) at 95% Confidence Interval (59.96-65.56%). Simple and mixed acid base disorders were observed in 332 (46.24%) and 386 (53.76%) patients respectively. Respiratory alkalosis was most common among 134 (40.36%) cases in simple acid base disorder whereas metabolic acidosis and respiratory alkalosis was most common among 204 (52.85%) in mixed acid base disorder. All types of disorders were observed more in elderly people (41-60 and >60 age group) than other age groups.

**Conclusions:**

Acid base disorder was found to be more common in very ill patients in emergency and intensive care units. Mixed acid base disorder was the most common with male and elderly patients in predominance.

## INTRODUCTION

Smooth physiological and well balanced functioning of a body depends on a very tight balance between the concentrations of acids and bases in the blood. Acid-base balance is important in the healthy maintenance of the cellular functions of the body. When there is imbalance between acid and base components in the body, it leads to Acid-Base Disorder (ABD). ABD is generally well correlated with high rates of morbidity and mortality.^1,2^

The appropriate diagnosis of ABD in critically ill patients requires measurement of plasma electrolytes and arterial blood gases by Arterial Blood Analysis (ABG), which evaluates metabolic and respiratory functions (pCO_2_, pH and pO_2_). An early diagnosis established by ABG can help in guiding the treatment of such patients and provide the details related with seriousness of the case.^3^ The main aim of this study was to find the prevalence of acid-base disorders and biochemical findings of such disorders in patients in a tertairy care hospital.

## METHODS

This is a descriptive cross-sectional study which was carried out between a periods of 1^st^ September 2018 to 31^st^ August 2019 at Nobel Medical College Teaching Hospital (NMCTH) after getting the approval from the Institutional Review Committee. All the patients, who presented to emergency department, Intensive Care Units (ICU), Neonatal ICU and in different wards undergoing ABG analysis in the department of Biochemistry, clinical laboratory services, were enrolled for the study. Whole sampling was done. All the participants had signed the informed consent for the study.

Arterial blood samples were collected from the patients presented in the different departments of NMCTH according to proper medical guidelines with all care on details such as site selection, collection procedures, sampling devices, sample handling etc. Sterile techniques were followed to prevent the site from being contaminated. Only those sample devices containing the proper amount of calcium-titrated heparin or lithium heparin as the anticoagulant were used to collect whole blood samples.

Arterial blood gas analysis was carried out by EDAN il5 blood gas and chemistry automated analyzer, which holds test cartridge in a portable and automated system that measures pH and blood gas, metabolites and electrolytes. It utilizes potentiometry and amperometry to determine the concentration of blood gas and blood chemistries. The test cartridge contains the fill port, the fluidic chamber, electrical contacts and an array of sensors. Different types of test cartridge contain different sensors.

Data were entered and calculations were done in Microsoft Excel, point estimate at 95% Confidence Interval was calculated along with frequency and proportion for binary data.

## RESULTS

**Figure 1:**
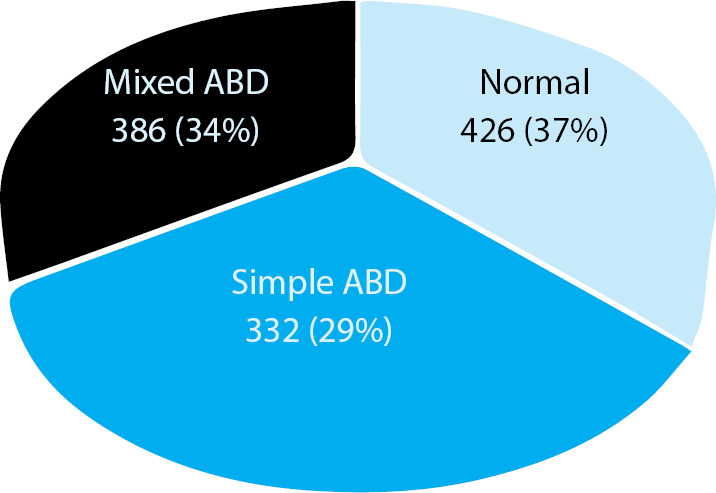
Distribution of patients.

A total of 1144 patients (admitted in Emergency department, ICU, NICU and in different wards) were evaluated for ABG analysis by sending their blood sample to the clinical laboratory services, NMCTH. Out of 1144 patients, the prevalence of acid base disorders was 718 (62.76%) at 95% Confidence Interval (59.96-65.56%). Simple and mixed acid base disorders were observed in 332 (46.24%) and 386 (53.76%) patients respectively (Figure 1). the ABD patients, we found that 403 (56.1%) were male and 315 (43.8%) were female.

**Figure 2:**
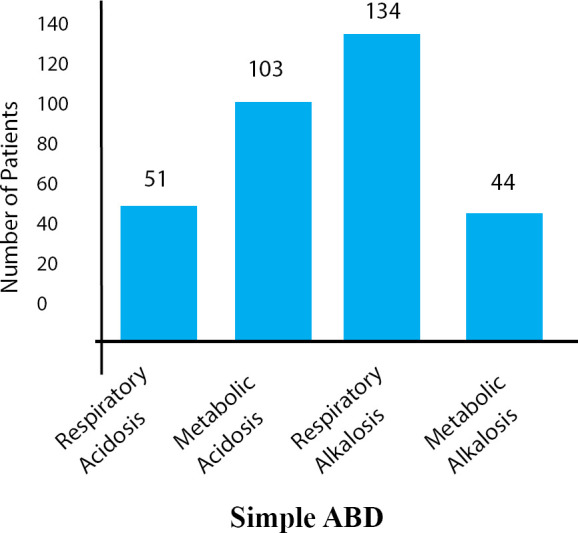
Distribution of Patients in simple ABD.

**Figure 3:**
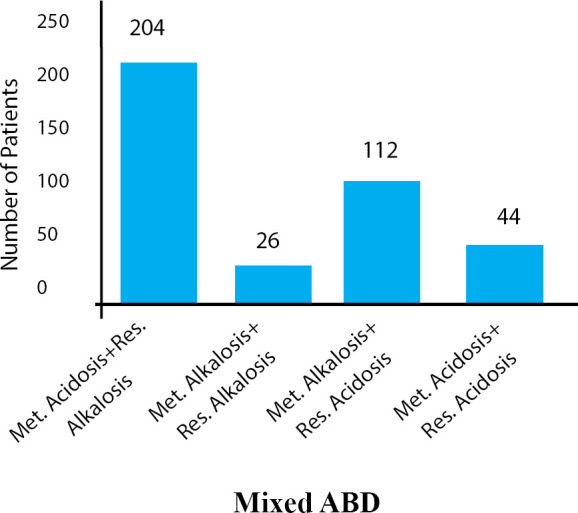
Distribution of Patients in mixed ABD.

Amongst the patients with simple ABD, metabolic acidosis was identified in 103 (14.3%), metabolic alkalosis in 44 (6.1%), respiratory acidosis in 51 (7.1%) and respiratory alkalosis in 134 (18.7%) (Figure 2). In the mixed ABD, it was noted that the maximum were suffering with metabolic acidosis and respiratory alkalosis 204 (28.4%). The other mixed ABD in our study were metabolic alkalosis and respiratory acidosis 112 (15.6%), metabolic acidosis and respiratory acidosis 44 (6.1%) and metabolic alkalosis and respiratory alkalosis 26 (3.6%) (Figure 3). The findings of biochemical parameters of ABD patients are mentioned (Table 1).

**Table 1:** Biochemical findings of ABD patients.

	Blood PH	PO2	PCO2	Bicarbonate	Sodium	Potassium	Calcium	Chloride	Lactate	Glucose
**Reference range**	7.38-7.42	80-100mmHg	35-45mmHg	22-26 mEq/L	135-146 mEq/L	3.5-5.0 mEq/L	1.15-1.30 mmol/l	98-106 mEq/L	0.5-1.0 mmol/l	4.0-7.8 mmol/l
**Simple Acid Base Disorder**										
**Met acidosis**	7.26±0.12	85.1±8.2	26.3±5.3	9.2±4.2	130.3±8.7	2.8±0.5	0.6±0.2	105.2±4.6	3.4±3.6	6.0±1.7
**Res acidosis**	7.32±0.14	92.8±14.6	72.6±8.6	34.2±3.2	141.4±3.6	4.0±2.6	0.8±0.08	84.2±10.7	3.6±4.2	6.5±1.1
**Met alkalosis**	7.55±0.05	109.2±17.9	58.7±9.5	67.0±12.5	130.3±2.6	2.7±0.5	0.7±0.2	62.3±7.1	1.3±0.8	10.7±6.3
**Res alkalosis**	7.50±0.07	96.1±13.5	12.3±6.1	27.2±5.5	125.3±6.4	2.9±0.4	0.7±0.3	124±22.6	2.2±0.9	8.4±3.5
**Mixed Acid Base Disorder**										
**Met acid+Res alk**	7.38±0.01	93.41±11.4	24.3±3.4	13.4±3.1	132.4±4.6	3.6±0.3	0.6±0.2	108.4±8.3	1.8±0.4	8.1±2.1
**Met alk+Res alk**	7.60±0.04	103.4±8.6	40.7±4.8	54.3±4.1	140.6±2.8	3.0±0.4	1.1±0.4	94.6±10.1	1.5±0.5	6.2±1.3
**Met alk+Res acid**	7.44±0.02	100.6±6.7	64.7±8.2	41.6±3.8	137.6±4.5	3.7±0.3	1.0±0.3	97.2±5.1	2.5±2.1	7.4±1.4
**Met acid+Res acid**	7.28±0.05	88.1±9.3	46.4±6.2	19.5±2.1	140.7±3.2	4.1±0.3	0.9±0.4	102.5±4.4	1.8±0.6	7.5±1.4

We have also evaluated our study by age group wise. Respiratory alkalosis was observed in 51 (7.1%) patients with age more than 60 years and in 43 (5.98%) patients with age 41-60 years. Metabolic acidosis, respiratory acidosis and metabolic alkalosis were also most commonly seen in more than 60 years in 38 (5.2%), 22 (3.0%), 19 (2.6%) patients respectively and in 41-60 years in 35 (4.9%), 18 (2.5%), 16 (2.2%) patients respectively. The data for number of patients suffering with simple ABD in other age group (Table 2). Mixed ABD was also mostly seen in higher age groups and the most common mixed ABD in our study population was metabolic acidosis and respiratory alkalosis, which was observed in 82 (11.4%) patients of more than 60 years age and in 68 (9.4%) patients of 41-60 age groups. Another major mixed ABD observed in our study was metabolic alkalosis and respiratory acidosis, which was seen in 41 (5.7%) and in 32 (4.4%) in >60 years age and 41-60 age group of patients respectively.

**Table 2:** Distribution of ABD patients by age wise.

Diagnosis	<20 Yrs	21-40 Yrs	41-60 Yrs	>60 Yrs
Respiratory Acidosis	5	6	18	22
Metabolic Acidosis	14	16	35	38
Respiratory Alkalosis	17	23	43	51
Metabolic Alkalosis	4	5	16	19
Met. Acid+Res. Alkalosis	29	25	68	82
Met. Alkalosis+Res. Alkalosis	3	4	8	11
Met. Alkalosis+Res. Acidosis	18	21	32	41
Met. Acidosis+Res. Acidosis	3	7	16	18
Total	93	107	236	282

## DISCUSSION

Acid base disorder is very common in critically ill patients and also strongly associated with mortality. Therefore, assessment of acid base status of such patients is an integral component of their treatment. Our findings have shown that the incidence of ABD in critically ill patients from the Emergency and other intensive care units of NMCTH is 63%. It can be compared with previous available reports, which elicits that the incidence rate of ABD in such patients in Korea,^4^ China,^5^ Italy,^6^ and Turkey^7^ is 66.4%, 94.2-97.3%, 56% and 71% respectively.

It was noted in our study that ABD was more common in male than female, which is similar to the findings reported earlier.^4,7^ In our study, it was seen that 29% of patients were suffering from simple ABD, whereas 34% of cases were of mixed ABD. In simple ABD cases, respiratory alkalosis was the most common but in mixed ABD, the maximum cases were of metabolic acidosis and respiratory alkalosis. Similar finding was observed in few studies, which reported as respiratory alkalosis as the most common type of simple ABD^8,9^ but Song ZF, et al.^5^ reported metabolic acidosis and respiratory alkalosis as not commonly observed in mixed ABD. In contrast to our results, a study carried out in China^5^ reported metabolic acidosis as the commonest simple ABD in sick people, whereas other studies conducted in USA^10^ and Scotland^11^ found metabolic alkalosis as the commonest type of simple ABD. Hodgkin JE, et al.^10^ reported metabolic acidosis and respiratory alkalosis (mixed ABD) as a less commonly observed ABD, which is different with our finding.

In our study, it was observed that ABD was more frequently seen in elderly patients with advanced age (>60 years). The reason for such a finding might be because of more incidence and severity of the diseases in advanced age group patients and they are also more likely to develop obstructive lung disease or kidney problem, which contributes the severity of ABD significantly. In addition to that, according to Nabata T, et al.^12^ various drugs and medication also affects acid-base status in advanced age patients.

The different biochemical parameters were estimated and were the basis of categorizing the cases as different types of ABD. The hallmark of metabolic acidosis was the decrease in blood pH and bicarbonate level with hypokalemia whereas for respiratory acidosis, it was decrease in blood pH with increase in pCO_2_ level and no change in sodium, potassium and chloride level. Similarly, the hall mark of metabolic alkalosis was increase in blood PH and bicarbonate level with hypokalemia, hyponatremia and hypochloremia, whereas increase in blood pH with decrease in pCO_2_ level with hypokalemia, hyponatremia and low ionized calcium were the hallmark for respiratory alkalosis. Similarly among the mixed ABD cases, normal blood pH, decrease pCO_2_, decrease bicarbonate with normal sodium, potassium and chloride level were the findings of metabolic acidosis and respiratory alkalosis. The findings of metabolic alkalosis and respiratory alkalosis were increase in blood pH, normal pCO_2_, increased bicarbonate, normal chloride, normal sodium and low potassium level. Nearly normal or slightly increased blood pH, increased pCO_2_, increased bicarbonate, normal or slightly decreased sodium, potassium level and normal chloride level were observed in metabolic alkalosis and respiratory acidosis. In case of metabolic acidosis and respiratory acidosis, decreased blood PH, normal or slightly increased pCO_2_, slightly decreased bicarbonate, normal sodium, potassium and chloride level were found.

## CONCLUSIONS

Acid base disorders are found to be the most common disorder among critically ill patients presented in the Emergency and other intensive care units. Therefore, the evaluation of arterial blood gas analysis becomes very important in understanding pathophysiology, making a diagnosis, planning effective treatment and monitoring progress. Mixed ABD was the most frequently observed case. Respiratory alkalosis was the most common among simple ABD case whereas metabolic acidosis and respiratory alkalosis was common in mixed ABD. Elderly people were more suffering with all types of ABD. Male suffered more from ABD than female.

## Conflict of Interest

**None.**
